# Zoomed out: digital media use and depersonalization experiences during the COVID-19 lockdown

**DOI:** 10.1038/s41598-022-07657-8

**Published:** 2022-03-10

**Authors:** Anna Ciaunica, Luke McEllin, Julian Kiverstein, Vittorio Gallese, Jakob Hohwy, Mateusz Woźniak

**Affiliations:** 1 Centre for Philosophy of Science, University of Lisbon, Campo Grande, 1749-016 Lisbon, Portugal; 2 Institute of Philosophy, University of Porto, via Panoramica s/n, 4150-564 Porto, Portugal; 3 Institute of Cognitive Neuroscience, University College London, London, WC1N 3AR UK; 4 University of Warwick, Coventry, UK; 5 Department of Cognitive Science, Social Mind Center, Central European University, Vienna, Austria; 6 Department of Psychiatry, Amsterdam University Medical Centre, Meibergdreef 5, 1105 AZ Amsterdam, The Netherlands; 7 Amsterdam Brain and Cognition, University of Amsterdam, Amsterdam, The Netherlands; 8 Department of Medicine and Surgery, Unit of Neuroscience, University of Parma, Via Volturno 39, 43121 Parma, Italy; 9 Cognition and Philosophy Lab, Department of Philosophy, Monash University, Melbourne, Australia; 10 Monash Centre for Consciousness & Contemplative Studies, Monash University, Melbourne, Australia

**Keywords:** Psychology, Health care

## Abstract

Depersonalisation is a common dissociative experience characterised by distressing feelings of being detached or ‘estranged’ from one’s self and body and/or the world. The COVID-19 pandemic forcing millions of people to socially distance themselves from others and to change their lifestyle habits. We have conducted an online study of 622 participants worldwide to investigate the relationship between digital media-based activities, distal social interactions and peoples’ sense of self during the lockdown as contrasted with before the pandemic. We found that increased use of digital media-based activities and online social e-meetings correlated with higher feelings of depersonalisation. We also found that the participants reporting higher experiences of depersonalisation, also reported enhanced vividness of negative emotions (as opposed to positive emotions). Finally, participants who reported that lockdown influenced their life to a greater extent had higher occurrences of depersonalisation experiences. Our findings may help to address key questions regarding well-being during a lockdown, in the general population. Our study points to potential risks related to overly sedentary, and hyper-digitalised lifestyle habits that may induce feelings of living in one’s ‘head’ (mind), disconnected from one’s body, self and the world.

## Introduction

The sense of self, the subjective experience of being an ‘I’ or a ‘self’ bound to my body as distinct from the world and others, is a key feature of our mental life^1–5^. Depersonalisation (DP henceforth) is a common dissociative experience characterised by profound alterations of one’s sense of self^6,7^. DP induces distressing feelings of being detached or ‘estranged’ from one’s self and body and/or one’s surroundings (derealisation): “I look in the mirror and it does not feel like myself I’m looking at. It’s like I’m floating, not actually experiencing the world, and slowly fading away into nothing. It’s like I’m on autopilot in somebody else’s body”^8^, P. 198.

This dramatic ‘split’ between the self, the body and the world is often reported by those with DP as ‘having a pane of glass’ interposed between one’s self, body and the world ^9,10^, affecting one’s sense of ‘realness’, i.e. the experience of being present and immersed in a real world here and now ^11^. Emotional flatness or numbness, as well as atypical emotional processing are core features of DP ^12,13^. Importantly, people experiencing DP report a significant impact of this self-detachment on the quality of their social and emotional life: “Feeling unreal and disconnected from my body and the world around me caused me to lose interest in the people and hobbies I used to love” ^8^, P. 190. These distressing feelings of being estranged from one’s self and ensuing social isolation and distancing from others^9,10^ is a common complaint among people experiencing DP.

In December 2019, the first cases of COVID-19 were reported in Wuhan, China and a state of pandemic was officially declared by the Emergency Safety Committee of the International Health Regulations. The COVID-19 pandemic presents us with a situation in which people are strongly encouraged to drastically change their lifestyle habits, to isolate themselves from others*,* and to maintain social distance. Now, experimentally induced social isolation and its impact on one’s self is ethically impossible to study in the lab. Yet, the current exceptional context allows examination ‘in the wild’ of whether lockdown-related changes affect people’s sense of self and presence in the world, and particularly feelings of DP.

Numerous studies suggest that these changes significantly impact people’s mental health worldwide^14–17^. The ensuing uncertainty and social distancing requirements have increased stress and anxiety levels in the general population^18,19^, and seems to aggravate symptoms for those with pre-existing mental health conditions^20^. For example, several studies ^21–23^ reported an increase of psychological distress, especially anxiety and depression during the COVID-19 lockdown. For example, Miguel-Puga et al.^24^ assessed the relationship between anxiety and depression symptoms and the development of post-traumatic symptoms in a population of frontline health workers. They reported that persistent burn-out may contribute to depersonalization symptoms and acute stress.

In the pre-pandemic context, DP had a prevalence of around 1–2% of the population^25–27^, which is comparable to, for example, schizophrenia^28^. Transient and mild episodes of DP are relatively common in the general population, often related to everyday phenomena such as fatigue^29^, sleep deprivation^30^, or travelling to new places^31^. Temporary occurrences of depersonalization episodes have been reported by almost 50% of college students^32–34^. DP typically co-occurs with highly traumatic events, or as a symptom of anxiety, panic, and depression^28,30^. Chronic states of depersonalisation and derealization symptoms may lead to a clinical diagnosis of Depersonalisation-Derealisation Disorder (DDD)^35^.

Despite the high prevalence, no study to date has examined the relationship between being estranged from *others* (due to events like COVID-19 lockdown restrictions), life habits changes, and experiences of DP. This gap is surprising because numerous pre-pandemic self-reports from people with DP point to the importance of maintaining close contact and proximal interactions with the physical environment and trusted others in their social environment in order to maintain one’s sense of self and presence: “When the depersonalisation is very deep, (…) it feels like that constant source of interaction is the only thing that allows me to maintain a connection with the world. I’ll also seek physical contact with whoever I’m with.” ^36^; “I really like when people scratch me or twist my arms or just touch me. It puts me back in my body and makes me feel cared for” ^8^, P. 87.

Previous work has emphasized the importance of dynamic and proximal embodied interactions with the physical and social environment on people’s sense of self and sense of presence in the world^37–40^. There is growing consensus that bodily self-awareness is not an awareness of the body in passive isolation from the physical and social world. Indeed, both classic phenomenologists such as Husserl^41^ and Merleau-Ponty^42^ and researchers working within cognitive science insist on the idea that bodily self-awareness ought to be understood primarily in relation to the environment, and to other people^37,43–48^. There is also growing evidence showing that multisensory integration of sensory signals arising from both inside and outside our bodies is fundamental to building a cohesive representation of our body, self and the world^49–52^.

Importantly, people experiencing DP report an inability to feel or judge where the body ends or begins; a lack of sensation or feedback from the body; inability to judge one’s position in space and one’s movements; or sensations of being divorced from one’s body ^7,9,28^. These alterations constrain people’s ability to feel fully present in their lives; to relate to others; and to participate in daily activities. It is crucial thus to address this question because, as one person with DP strikingly puts it: “a disorder that makes you feel invisible is invisible in society” ^8^, P. 193.

We conducted an online study to investigate the relationship between digital media-based activities and social interactions, and peoples’ sense of self during the lockdown as contrasted with before the pandemic.

The exceptional COVID-19 context triggers simultaneously a sharp (a) *decrease* in dynamical and proximal interactions with the environment (people move less, interact less and touch less the surroundings and others); (b) and *increase* in digital and distal media interactions (where audio-visual modalities are predominant, and the bodily movements and activities are more limited)^53^. Use of digital multimedia technologies has become an important part of modern life, notably as a marker of community integration, allowing instantaneous communication, status updates, and social networking among individuals^54,55^. These digital platforms enable users to establish digital connections to other users via (1) text messaging on cellular phones, (2) social networking sites such as Facebook, Twitter, Instagram, (3) social e-meetings platforms such as Zoom, Teams, etc.; and (iv) online videogaming.

We set out to examine whether: (a) people who spend more time using indoors digital, technology-based platforms for activities and social interactions during the lockdowns may also show increased DP experiences; (b) people who spend more time doing outdoor physical activities (e.g. walking, running, playing football, etc.), as well as manual work involving rich multisensory experiences (such as gardening, cooking, pottery, etc.), may show decreased DP experiences. (c) In addition, we aimed at examining what aspects of our self-experiences and activities were most impacted during these long episodes of radical lifestyle changes and social distancing. (d) Finally, we also looked at the impact of these lockdown related changes on the perceived vividness of our positive and negative emotions.

## Results

### Lifestyle habits during the COVID-19 lockdown

**Figure 1:**
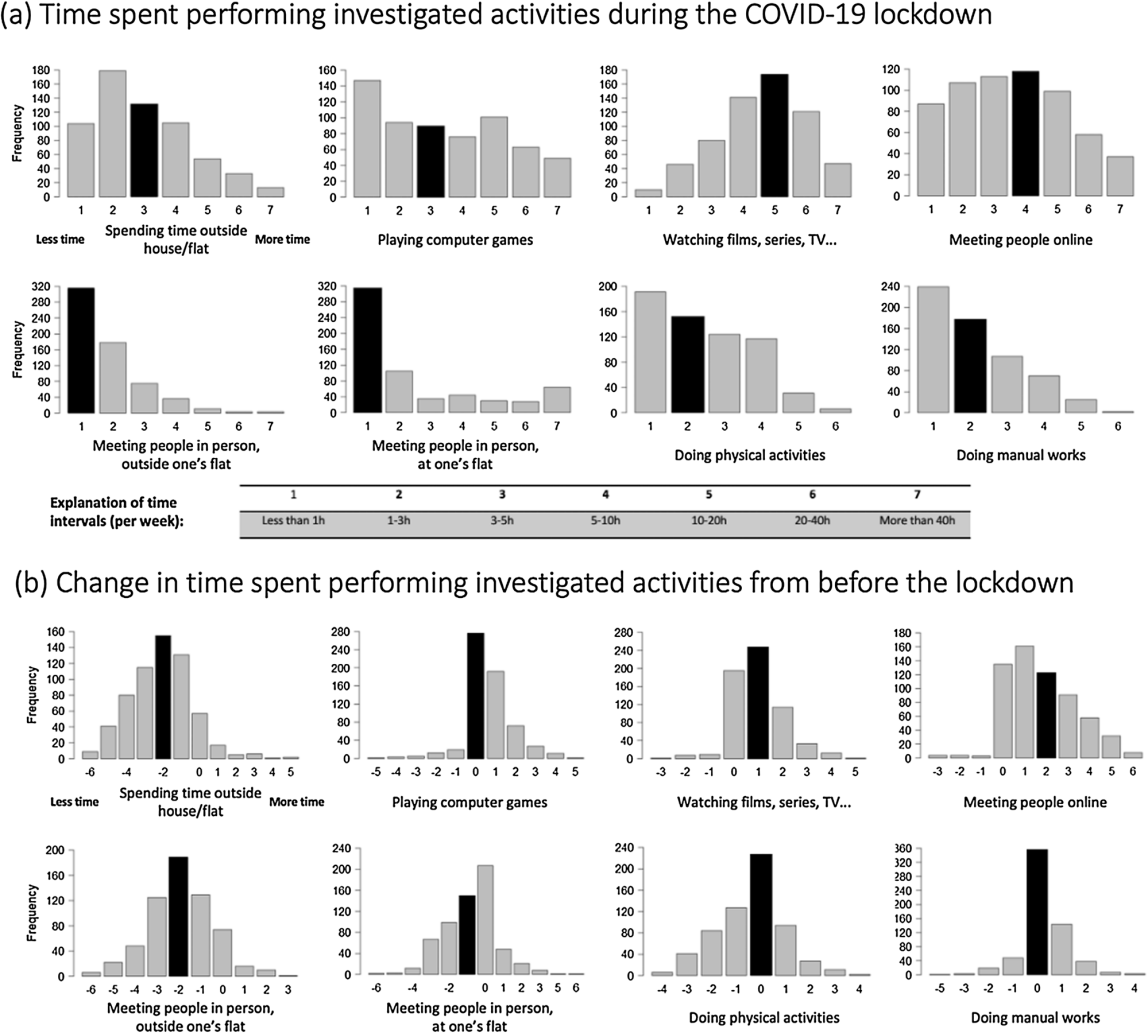
Histograms displaying distribution of weekly time spent performing investigated activities during lockdown (top panels) and the change in time spent doing these activities between lockdown and before the COVID-19 pandemic started (bottom panels). The change in time spent is indicated in the number of categories that changed—negative values indicate spending less time on a given activity during the lockdown than before, and positive values the opposite. Black bars indicate median values.

Participants reported spending most time: (a) watching non-interactive audiovisual media (movies, series, TV, internet videos) with the median indicating that a typical participant was spending 10–20 h weekly on this activity (Fig. 1); (b) participating in online meetings (median 5–10 h/week); (c) playing computer games (median 3–5 h/week, but with a very balanced distribution of responses across participants) and (d) spending time outside one’s place of residence (median 3–5 h/week). Participants reported a median of 1–3 h/week) doing physical exercises and doing manual work, and less than median 1 h/week meeting people outside or inside their place of residence.

When comparing participants’ lifestyle habits during the lockdown and before the pandemic, the highest increase was in the time spent participating in online meetings (W(621) = 2534, p < 0.001; median + 2 categories: from 1–3 h to 5–10 h/week), and watching non-interactive audiovisual materials (W(620) = 4097, p < 0.001; median + 1 category: from 5–10 h to 10–20 h/week). A smaller increase (not affecting the median) was also observed for playing computer games (W(621) = 8498, p < 0.001), and doing manual activities (W(620) = 9857, p < 0.001). By contrast, the largest decrease of activity was in the amount of time spent outside of one’s place of residence (W(620) = 1.5 + e6, p < 0.001; median—2 categories: from 10-20 h to 3-5 h/week), meeting people outside one’s home (W(621) = 1.4 + e6, p < 0.001; median—2 categories: from 3–5 h to less than 1 h/week), and meeting people at one’s flat or house (W(621) = 7.2 + e5, p < 0.001; median—1 category: from 1–3 h to less than 1 h). A smaller decrease in activity (W(621) = 5.5 + e5, p < 0.001; not affecting the median) was present for doing physical activities.

To further investigate the pattern of lifestyle habits during the lockdown we conducted two principal components analyses (PCA) (see Table 1). The first PCA (A in Table 1) encompassed responses regarding frequencies of performing specific activities during the lockdown. Our analysis (model’s Chi^2^ (13) = 304.3, p < 0.001, Oblimin rotation, minimal correlation for factor loading r = 0.4) revealed that lifestyle habits investigated in our study comprised two independent factors (correlation between factors r = 0.08). The first factor consisted of items (all of them were positive factor loadings): (a) spending time outside home, (b) meeting people outside home, (c) physical activity, and (d) manual work. The second factor consisted of items: (e) playing computer games, (f) watching TV and audiovisual materials, and (g) participating in online meetings (also all responses were positive factor loadings). The results of the analysis revealed that interacting with people at one’s home did not contribute to any of these factors.

**Table 1:** Results of two principal components analyses (factor loadings and uniqueness) performed on the results of the lifestyle habits survey.

Activity	Factor 1	Factor 2	Uniqueness
**(A) PCA of time spent on activities during the lockdown**			
Spending time outside one’s flat/house	0.73		0.468
Playing computer games		0.727	0.467
Watching movies, TV, series etc.		0.447	0.782
Meeting people online		0.704	0.476
Meeting people in person outside	0.622		0.538
Meeting people in person at home			0.849
Doing physical activity	0.739		0.456
Doing manual work	0.512		0.726
**(B) PCA of change in doing activities before/during lockdown**			
Spending time outside one’s flat/house	0.686		0.52
Playing computer games	− 0.427		0.818
Watching movies, TV, series etc.	− 0.566		0.654
Meeting people online	− 0.541		0.635
Meeting people in person outside	0.758		0.42
Meeting people in person at home			0.858
Doing physical activity		0.694	0.469
Doing manual work		0.779	0.376

The second PCA (B in Table 1) was conducted on differences between lifestyle habits before and during the pandemic. The second PCA (model’s Chi-squared(13) = 327.1, p < 0.001, Oblimin rotation, minimal correlation for factor loading r = 0.4) revealed a different factorial structure than the first one. The first extracted factor shows that people who were spending *less* time outdoors than before the pandemic were also (a) meeting relatively less people outside one’s flat (than before the pandemic) and were (b) more likely to report playing more computer games, (c) watching more audiovisual materials and (d) participating more in online meetings. This factor indicates that the more people were restricting outdoors activities, the more they engaged in media consumption and digital social interactions. The second factor consisted of two items which both positively loaded the factor: (a) physical activity and (b) manual work, indicating that people who started performing more physical activity during the lockdown were also more likely to start performing more manual work. We also found the opposite: people who were doing less physical activity during the lockdown were also less likely to do manual work. The two extracted factors did not correlate with each other (r = 0.003). Once again, social interactions in person at one’s home did not load any of the extracted factors.

Finally, we investigated how strongly each of the activities measured in our survey was related to the overall perception that lockdown influenced one’s life. We have thus conducted a non-parametric correlation analysis between the estimated influence of lockdown on one’s life and time spent weekly on the behaviours our survey measured. The results revealed (Table 2) that the perceived influence of lockdown was significantly related to the change in performed activities before and during the lockdown for all activities included in our survey, except for manual work. The more people felt the influence of lockdown on their lives, (1) the *less* time they were spending being outside their flat/home, meeting people outside their flat/home, meeting people in their flat/home, and doing physical activities; and (2) the *more* time they spent watching audiovisual materials, participating in online meetings, and playing computer games.

**Table 2:** Correlations between lifestyle habits and general perception that lockdown influenced one’s life. *** = significant at the 0.001 level, ** = significant at the 0.01 level, and * = significant at the .05 level

Activity	Tau B		p	BF10
Spending time outside one’s flat/house	− 0.234	***	< 0.001	2.4e + 15
Playing computer games	0.127	***	< 0.001	5776
Watching movies, TV, series etc	0.218	***	< 0.001	1.4e + 13
Meeting people online	0.188	***	< 0.001	2.540e + 9
Meeting people in person outside	− 0.203	***	< 0.001	1.696e + 11
Meeting people in person at home	− 0.202	***	< 0.001	1.241e + 11
Doing physical activity	− 0.097	**	0.003	37.368
Doing manual work	0.04		0.249	0.159

### Relationship between depersonalization and lifestyle habits during lockdown

We conducted a correlational analysis (uncorrected for multiple comparisons) between the scores on the Cambridge Depersonalisation Scale (CDS-29)^56^ (see “Methods” section below) and (a) responses to questions pertaining to participant’s lifestyle habits now; (b) scores reflecting how participant’s lifestyle habits have changed as a result of the pandemic. Table 3 presents the results of this analysis.

**Table 3:** Correlations between depersonalization and lifestyle habits. *** = significant at the 0.001 level, ** = significant at the 0.01 level, and * = significant at the .05 level

Correlation with depersonalization (total score in CDS-29)					
Item	Tau B		p	BF10	BF01
Overall influence of lockdown on life	0.151	***	< 0.001	4.20E + 05	2.3e − 6
**Activities during lockdown**					
Spending time outside one’s flat/house	− 0.03		0.3	0.1	9.9
Playing computer games	0.1	***	< 0.001	55.8	0.018
Watching movies, TV, series etc.	0.026		0.383	0.08	12.1
Meeting people online	0.077	**	0.008	3.2	0.32
Meeting people in person outside	− 0.009		0.771	0.056	17.9
Meeting people in person at home	− 0.002		0.947	0.053	18.9
Doing physical activity	− 0.066	*	0.027	1.073	0.93
Doing manual work	0.043		0.15	0.195	5.1
**Change in doing activities in relation to before the lockdown**					
Spending time outside one’s flat/house	− 0.019		0.51	0.068	14.7
Playing computer games	0.138	***	< 0.001	27,880	3.6e − 5
Watching movies, TV, series etc.	0.109	***	< 0.001	194	0.005
Meeting people online	0.054		0.063	0.41	2.4
Meeting people in person outside	− 0.015		0.606	0.062	16.2
Meeting people in person at home	0.023		0.429	0.077	13
Doing physical activity	− 0.027		0.354	0.089	11.3
Doing manual work	0.022		0.476	0.074	13.6

Firstly, we found that the subjective estimation of how strongly lockdown is felt to have influenced one’s life was correlated with the occurrence of DP experiences. Specifically, participants who reported that lockdown had strongly influenced their life also had higher scores on the CDS-29.

We also found that higher DP was positively related to digital media-based activities and distal social interactions. For example, participants who were spending more time during lockdown playing computer games, and who reported that they increased the time that they spent playing computer games during the lockdown (as compared to the pre-lockdown period) reported significantly stronger DP symptoms. Secondly, consumption of passive media such as watching movies, YouTube videos and television did not positively correlate with DP by itself, but people who reported that they were spending more time doing this during lockdown than before showed increased DP. Finally, frequent participation in online meetings was positively correlated with DP. Moreover, we found a weak significant negative correlation between the frequency of physical exercises during lockdown and the magnitude of DP showing that physical exercise during lockdown was weakly correlated to lower level of DP.

In contrast to digital media consumption, the amount of in person social contact outside of the home did not influence DP. Neither the absolute time spent meeting with other people indoors and outdoors, nor the difference in these activities before and after the lockdown, correlated with DP, with Bayesian analyses showing strong support for a null hypothesis.

Finally, with regards to the vividness of participants' emotions, we found that change in vividness of experienced emotions was significantly related to the occurrence of DP experiences. People with higher DP scores experienced positive emotions less vividly (Tau B = − 0.21, p < 0.001, BF10 = 2.2e + 11) and negative emotions more vividly (Tau B = 0.19, p < 0.001, BF10 = 4.1e + 9) than people with lower occurrences of DP experiences.

## Discussion

This study examined the relationship between lockdown-related restrictions and experiences of DP during the lockdown as contrasted with before the pandemic. DP is a common dissociative phenomenon that makes people feel ‘estranged’ and detached from themselves and from their bodies^6^,which in turn may lead to estrangement from others and consequent social isolation^9,10^.

Our study revealed several key findings. Firstly, we found that an increased use of digital media-based activities (especially playing computer games) correlated with higher feelings of DP. Strikingly, increased participation in online social e-meetings (as contrasted with the pre-lockdown period) was also positively correlated with DP experiences. We also found that people who reported that they were spending more time during the lockdown engaged in sedentary digital media-consumption such as watching films, TV, YouTube videos (as contrasted with the pre-lockdown period), also showed DP experiences. Moreover, people reporting higher experiences of DP in our survey also reported enhanced vividness of negative emotions (as contrasted with positive emotions). Our study also reveals a weak significant negative correlation between the frequency of physical exercise during the lockdown and the occurrence of DP experiences. Intriguingly, the amount of in person social interactions, both indoors and outdoors, did not influence DP. Finally, our results suggest that the subjective estimation of how strongly lockdown restrictions influenced one’s life was related to the occurrence of DP experiences. Specifically, participants who reported that lockdown influenced their life to a great extent had higher scores on the CDS-29. Below we discuss these findings in detail.

One of our key findings was that participants who (a) either spent a lot of time playing video games in general, or (b) reported that during the lockdown the time they spent playing video games had increased as compared to before the lockdown, showed higher depersonalisation experiences according to the CDS-29. While the direction of causation between these two phenomena is currently an open question, one may speculate about potential explanatory factors. For example, one may argue that computer-based gaming involves restricted and repetitive proprioceptive and proximal tactile stimulation (e.g. fingers on the keyboard, the mouse, or the telecommand, feet on the floor, sedentary posture, etc.). Indeed, videogaming, as the label suggests, rests heavily on enhanced visual and auditory sensory stimulation, to the detriment of proprioceptive, interoceptive, olfactory and tactile stimulation. This imbalance towards exteroceptive stimuli (being overstimulated by the outside or the ‘virtual’ world ‘out there’ may explain why people who spend more time videogaming also report higher feelings of self-detachment and disembodiment, as well as lack of presence in the real world ‘here and now’. Moreover, previous research has also shown the critical importance of dynamic, embodied and multisensory interactions with the social and physical environment for one’s sense of self and mental health^40,46,52,57^.

Another potential interpretation is that by disengaging from bodily signals, people may become more tempted to get more attentionally and emotionally involved in virtual realities than the real one. For example, playing computer games involves attentional detachment from both physical reality in favour of immersion in a virtual one, as well as detachment from one’s body in favour of embodiment of one’s digital body—an avatar^58,59^. Previous work outlined that the sense of self is critically dependent on bodily (i.e., interoceptive and proprioceptive) sensory processing^1,2,39,60,61^. If this is so, then distracting oneself from one’s somatic feelings via digital audiovisual materials that puts the body in a more passive setting, should lead to higher DP. By contrast, higher dynamical and embodied engagements with the surroundings should lead to lower DP. Our findings seem to provide strong support for the former hypothesis and weak significant support for the latter hypothesis. Indeed, we found a weak significant negative correlation between the frequency of physical exercise during the lockdown and the occurrence of DP experiences. This suggests that physical exercise (i.e. dynamic activity engaging the entire moving body) may be related to lower DP. This idea is in line with previous work showing that DP is underpinned by disrupted bodily sensory processing as demonstrated by several studies^62–66^.

Another important finding is that increased participation in online and distal social e-meetings (as opposed to pre-lockdown period), positively correlated with higher DP experiences. The absolute time spent on e-meetings during the lockdown was positively related to increased DP, while the difference between lockdown and pre-lockdown did not reveal any effect. Again, while the direction of the causation between these phenomena remains to be further investigated, here we can speculate in favour of the impact of diminished dynamic and multisensory, proximal interactions with the physical and social environment on one’s sense of self. During technology-mediated social interactions the body posture is primarily sedentary. Moreover, the communication is primarily audiovisual in a flat two-dimensional space, lacking the richness of the multisensory cues and affective reward that people typically gain from face-to-face in person interactions. Enhanced use of audiovisual signals situated ‘out there’ may prompt people to disengage, detach and pay less attention to bodily signals from ‘here’, that is inside and closer to the physical body^39,40^.

Given that humans are highly social beings, one may find surprising our results suggesting that online social interactions enhance feelings of DP. For example, recent work exploring the link between loneliness, social isolation and social media provides a rich and more nuanced picture, with studies showing the importance of maintaining social media connectedness for people’s well-being^67–71^. The relationship between social connectedness and social internet use is currently an open question, and further work is needed to carefully investigate the multifaceted aspects of these digital interactions. However, here we speculate in favour of the hypothesis that what matters for human wellbeing may not be just any type of social interactions, but *close* social interactions, that is, staying in touch (literally) with trusted and loved ones. This hypothesis seems to receive further support from recent studies exploring the relationship between social touch deprivation and psychological wellbeing during COVID-19^17^. Indeed, in early life and beyond, humans actively meet other peoples’ bodies (via skin-to-skin interactions) before they meet others’ minds and interact with them from an visuo-distal viewpoint on a screen^39,40,46,48,52,72^. Yet, during the lockdown, and because of the social/physical distancing constraints, people spent a significantly higher amount of time in work-related distal e-meetings. Moreover, some people drastically reduced their face-to-face social interactions outside one’s household, reducing thereby the richness of their social life. While some of those who experienced the lockdown with their close family may have benefited from the increase of amount of time spent together, by contrast, those who had conflictual family relationships may have benefited less from the indoors social interactions, putting them at risk. This may potentially explain our intriguing results highlighting a positive correlation between increased online e-meetings use and higher DP. Further work needs to disentangle these aspects in a systematic manner.

Another interesting result of our study is that people reporting higher experiences of DP in our survey also reported enhanced vividness of negative emotions (as opposed to positive emotions), which can be linked to higher anxiety and stress due to the uncertainty and threat posed by the COVID-19 pandemic. This is in line with previous work suggesting that a core feature of DP is atypical emotional processing: “I don’t have any emotions- it makes me so unhappy”^12,62^. As Medford notes, this may seem self-contradictory, but on further questioning, the person explained that he experienced considerable *inner* turmoil, but felt reduced emotional response to *external* events or other people. Again, this is in line with the idea that DP involves feelings of being simultaneously ‘trapped’ in one’s mind, and outside one’s body, with enhanced focus on inner workings to the detriment of active and affective responsiveness to the external environment^48,73,74^.

Intriguingly, the number of in person social interactions, both indoors and outdoors, did not influence depersonalization. This may be due however to a potential flooring/ceiling effect, as people were explicitly instructed to avoid in-person social interactions as much as possible. Indeed, given that (a) the largest decrease of self-reported activity during the lockdown (as opposed to pre-pandemic) was the time spent meeting people in person outside or inside one’s home, and (b) we do not know to what extent these indoors and outdoors meetings involved close, touch-based social interactions (e.g. hugging vs saying ‘hello’ from safe distance), the relationship between social contact and DP remains inconclusive at this stage. Further work is needed to disentangle different aspects of social experiences (distal vs proximal) and DP.

It is likely that at the time of administering our questionnaire (23 April and 8 May 2021) all our participants had been significantly impacted by a lockdown in the last six months regardless of their place of residence (we have excluded those participants that reported not having experienced any lockdown in the past 6 months). However, different European countries imposed different lockdown measures (especially the Northern versus the Southern ones). In order to verify potential differences between participants’ countries with regards to reported impact of lockdown on their lives we carried out an additional analysis (see Supplementary Material 1 and Supplementary Table 1) This analysis revealed no significant differences between any of the countries used in the analysis (we took the top six countries with respect to country of residence) with respect to the reported impact of lockdown on their lives. Thus, we are confident that our effects are down to the impact that the lockdown had on our participants on the individual level, rather than any between country differences with respect to the perceived impact of lockdown on everyday life.

Finally, our results suggest that the subjective estimation of how strongly lockdown restrictions influenced one’s life was related to the occurrence of DP experiences. Specifically, participants that reported that lockdown influenced their life to greater extent, had higher scores in CDS-29. Although our study did not involve a systematic comparison of the same individuals before the pandemic and during its course (see “Limitations” section below), our findings strongly suggest that the experience of lockdown also led to increase in DP experiences. Indeed, lockdown-related restrictions led to a widespread increase in anxiety and stress^23,23,75^, which, combined with lack of proximal social interactions and increased digital-media consumption, may lead to DP experiences. On the other hand, since this study does not determine the direction of causation, it is also possible that people with higher DP experiences might be simply more likely to be sensitive to lockdown-related changes and hence report them as more strongly affecting this life. If the second hypothesis is correct, then on average participants tested during the lockdown should not differ in the occurrence of DP from people tested before the COVID-19 pandemic.

Although our study did not directly investigate this issue, our sample exhibited much higher DP scores compared to what has typically been observed in samples obtained before the COVID-19 lockdown. For example, Aponte-Soto et al.^76^ published a study with 300 Italian participants where they reported an average general score in CDS-29 of *M* = 16.3 (*SD* = 18.3, range = 0–132). They also reported that only 2% (6 participants) obtained a score of ≥ 70 indicating, according to them, clinically significant DP symptoms. In contrast, in our study the average score was much higher and reached *M* = 49.3 (*SD* = 35.9, range = 0–244), and almost a quarter of participants (24%) obtained clinically significant scores ≥ 70. Other, smaller studies reported similar values to Aponte-Soto et al.’s. Sugiura et al.^77^ in a Japanese version of CDS-29 tested on a Japanese population found that the average score in a control group that was not diagnosed with DP was *M* = 15.1 (*SD* = 16.5). Zingrone et al.^78^ conducted another two large questionnaire studies with CDS-29 and found a relatively large average score on CDS-29 in their samples: Study 4: n = 256, *M* = 20.1, *SD* = 19.4, range = 0–93; Study 5: n = 591, *M* = 37.2, *SD* = 14.2, range = 0–279. However, while Study 4 was conducted on a random sample of a small city in central Virginia, USA, study 5 was conducted on subscribers of a para-psychology email newsletter. However, people interested in parapsychology might have the tendency to report more uncommon experiences than the rest of the population. Thus, the results from Zingrone and colleagues’ study 5 might reflect elevated average DP experiences compared with the rest of the population.

More indirect evidence in support of the idea that lockdown-related experiences may lead to DP comes from the finding that in our sample the correlation between the CDS-29 subscales was higher than in previous studies. This suggests a more homogenous and global phenomenon, consistent with the fact that a significant amount of people worldwide experienced similar restrictions and ensuing negative states due to the pandemic. Overall, while our study does not provide direct ultimate evidence that COVID-19 lockdown caused an increase in the occurrences of experiences of DP, it provides indirect support for a causal link when our results are compared with the results of previous studies that used CDS-29.

### Limitations and outlook

There are several important limitations of our study. Ideally, the same participants who took part in our online study should have been assessed for their DP experiences *before* the pandemic. Moreover, asking participants to recall their lifestyle habits from before the pandemic (probably six to nine months before the survey) is likely to be less accurate than if we had actually asked them about these habits before the pandemic (i.e., before March 2020). However, the occurrence of the COVID-19 pandemic was unforeseeable, hence our study was tailored ‘on the fly’ to assess lockdown-related experiences pre- vs during the pandemic as self-reported *during* the pandemic. Further work is needed to contrast DP experiences before and during the pandemic in the same large sample of participants.

Second, our study was conducted between April and May 2021, and both the CDS-29 and our tailored lockdown survey were asking participants about their experiences in the previous 6 months. This means that participants were describing their lives during late autumn, winter, and early spring, as most of our participants were European-based. It is possible that life activities reported by participants were influenced by the fact that participants were asked about a period of the year in which they generally spend more time indoors (i.e., compared to summer). However, this should not affect correlations with CDS-29. Also, as we mentioned earlier, our study did not collect specific data about the type of in person interactions (distal versus proximal, romantic versus friends) that people had both indoors and outdoors during the pandemic.

Finally, we acknowledge that the use of an online recruitment tool (due to resource limitations) may limit the generalizability of our findings because demographic groups are unequally represented (e.g., an underrepresentation of those with limited computer use) due to coverage bias^79^. Future studies may employ probability sampling methods in order to address this topic in a more representative sample.

Our study may help to tackle key questions related to human well-being in the general population during a lockdown. Our results suggest that paradoxically, increasing online social interactions and digital activities may have negative effects in some people, such as inducing feelings of living more in one’s ‘head’ (mind) and less in one’s body. These findings point also to potential risks related to overly sedentary and hyper-digitalized lifestyle habits that may make people feel less ‘real’ and less connected with their close physical and social environment.

## Methods

### Participants

An a priori Power analysis conducted using G*Power 3.1.9.2 revealed that in order to detect correlations equal to or stronger than rho = 0.15 with alpha = 0.05 and beta = 0.95 the required sample size needs to be at least 571 participants. We estimated our target sample size to be approximately 640 people.

We recruited 691 people from the Prolific crowdsourcing platform (www.prolific.co). Data collection took place between 23 April and 8 May 2021. For the analysis of effects of COVID-19 lockdown we excluded participants that reported that they hadn’t experienced lockdown during the last 6 months for more than one month and those that left this question unanswered (69 people in total). Because we assumed that those who did not finish the survey (46 people) had also revoked their consent for us to use their data, their responses were not recorded.

Our final sample consisted of 622 participants. The mean age of all participants was 31.5 years (SD = 12.2, Median = 27, range 18–78). 257 of participants were female, 358 were male and 74 did not disclose their gender. All participants were required to be fluent in English (with respect to the participants’ reported language proficiency on Prolific). Participants represented predominantly European nationalities: UK (220 participants), Poland (93), Portugal (65), USA (34), Italy (33), the Netherlands (22), Greece (18), and Germany (10). The remaining participants represented diverse nationalities from around the world. 73 participants did not disclose their nationality on Prolific. The places of residence of participants were predominantly European countries (550 participants), with the exception of Australia (1), Canada (7), Chile (1), Israel (3), Mexico (5), South Africa (18) and USA (33). 71 participants did not indicate their country of residence on Prolific. We did not place any constraints on the participants’ place of residence or birth, therefore the distribution of our sample with regards to these was down to chance.

Informed consent was obtained from all participants before the start of the experiment according to procedures approved by the Monash University Human Research Ethics Committee (MUHREC). The experiment was conducted in accordance with the Declaration of Helsinki.

### Measures

#### Cambridge Depersonalization Scale

All participants first completed the Cambridge Depersonalization Scale (CDS-29)^54^. CDS-29 is a 29 items standard questionnaire used to evaluate the severity of occurrence of depersonalization experiences by asking participants to estimate their frequency (“never”, “rarely”, "often", "very often", "all the time") and duration ("for a few seconds", "for a few minutes", "for a few hours", "for about a day", "more than a day", "more than a week") in the past six months. The total score (between 0 and 290) points was calculated by summing over all items. The CDS-29 has good statistical properties^56,76,77,80,81^ with internal reliability for different language versions reported between 0.89 and 0.94 (Cronbach’s alpha). Moreover, previous research has extracted four subscales from CDS-29^82^: Anomalous Body Experience, Emotional Numbing, Anomalous Subjective Recall, and Alienation from Surroundings. Given that in our sample all of these subscales correlated with each other more strongly (correlations between factors from r = 0.61 to 0.66) than in Sierra et al.^80^ who reported correlations between r = 0.23 and 0.34, we decided to use only the full total score.

**Figure 2:**
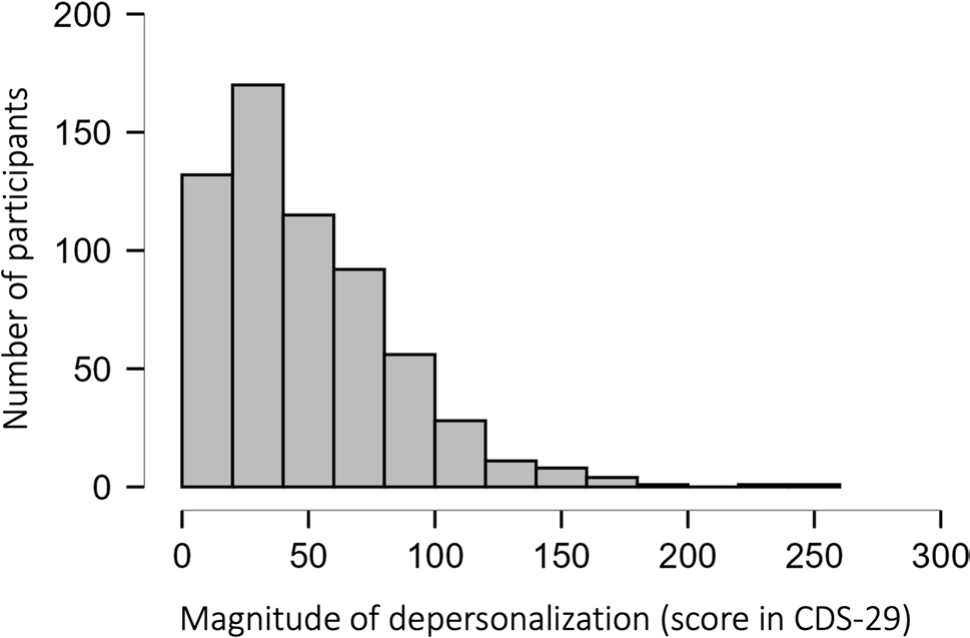
Distribution of total scores in CDS-29 in our sample.

The average total score for the CDS-29 in our sample was 49.3 (SD = 35.9), with median at 41 points, mode at 32 points, and range between 0 and 244 points (with theoretical maximum being 290 points). The distribution of scores was strongly right-side skewed (Skewness = 1.29) and slightly leptokurtic (Kurtosis = 2.85) reflecting the fact that the majority of participants reported relatively low scores (the middle 50% of participants had scores between 23 and 68) (see Fig. 2). An investigation into the psychometric properties of our CDS-29 scores revealed that internal reliability was similarly high to previous studies, with Cronbach’s alpha = 0.93. However, principal components analysis (PCA) revealed a different factorial structure than previous analyses. This was also reflected by relatively high correlations between subscales as determined by ^80^, which varied between Pearson’s r = 0.62 and 0.66. Due to this, all of our reported analyses were conducted on the total score rather than the subscales.

#### COVID-19 lifestyke habits survey

(2) The second measure used in our study was a custom-designed lifestyle survey asking about life activities before and during the COVID-19 pandemic. Table 4 presents the full list of questions. First, participants were asked whether they experienced COVID-19 related lockdown during the last 6 months (the same duration covered by the CDS-29). We included this question in order to screen out participants that were not affected by lockdown. The second question asked participants to evaluate to what extent they felt that the lockdown influenced their life on a 5-point Likert scale. In the main part of the survey, participants were asked to separately evaluate how many hours per week they used to spend (a) before the pandemic and (b) during the last 6 months (during the pandemic) doing each of eight activities presented in Table 4. The questions were selected based on our research hypotheses regarding what activities might influence depersonalization, such as in-person versus digital social activities, as well as physical activities versus media use. Finally, at the end of the survey participants were asked about how the COVID-19 lockdown influenced the vividness of their emotional experiences, separately for positive and negative emotions.

**Table 4:** Questionnaire measuring lifestyle habits before and during the lockdown.

**Have you experienced COVID pandemic-related restrictions (lockdown) during the last 6 months for more than 1 month?**								
Yes			No					
**To what extent have pandemic-related restrictions influenced your life?**								
1: Not at all	2	3			4	5: Very strongly		
**How many hours per week were you spending outside of your house/flat:**								
Before the pandemic	Less than 1 h	1–3 h		3–5 h	5–10 h	10–20 h	20–40 h	More than 40 h
During the last 6 months	Less than 1 h	1–3 h		3–5 h	5–10 h	10–20 h	20–40 h	More than 40 h
**How many hours per week were you spending playing computer games:**								
Before the pandemic	Less than 1 h	1–3 h		3–5 h	5–10 h	10–20 h	20–40 h	More than 40 h
During the last 6 months	Less than 1 h	1–3 h		3–5 h	5–10 h	10–20 h	20–40 h	More than 40 h
**How many hours per week were you spending watching films, series, TV programs and other videos (Youtube videos, etc.)**								
Before the pandemic	Less than 1 h	1–3 h		3–5 h	5–10 h	10–20 h	20–40 h	More than 40 h
During the last 6 months	Less than 1 h	1–3 h		3–5 h	5–10 h	10–20 h	20–40 h	More than 40 h
**How many hours per week were you spending meeting with other people online (video-chatting via virtual platforms such as Zoom, Microsoft Teams, Skype, etc.)**								
Before the pandemic	Less than 1 h	1–3 h		3–5 h	5–10 h	10–20 h	20–40 h	More than 40 h
During the last 6 months	Less than 1 h	1–3 h		3–5 h	5–10 h	10–20 h	20–40 h	More than 40 h
**How many hours per week were you spending meeting with other people in person OUTSIDE your flat/house**								
Before the pandemic	Less than 1 h	1–3 h		3–5 h	5–10 h	10–20 h	20–40 h	More than 40 h
During the last 6 months	Less than 1 h	1–3 h		3–5 h	5–10 h	10–20 h	20–40 h	More than 40 h
**How many hours per week were you spending meeting with other people in person IN your flat/house (including the people that you live with)**								
Before the pandemic	Less than 1 h	1–3 h		3–5 h	5–10 h	10–20 h	20–40 h	More than 40 h
During the last 6 months	Less than 1 h	1–3 h		3–5 h	5–10 h	10–20 h	20–40 h	More than 40 h
**How many hours per week were you spending doing physical activity**								
Before the pandemic	Less than 1 h	1–3 h		3–5 h	5–10 h	10–20 h	20–40 h	More than 40 h
During the last 6 months	Less than 1 h	1–3 h		3–5 h	5–10 h	10–20 h	20–40 h	More than 40 h
**How many hours per week were you spending doing manual works (for example: gardening, painting, pottery, playing an instrument):**								
Before the pandemic	Less than 1 h	1–3 h		3–5 h	5–10 h	10–20 h	20–40 h	More than 40 h
During the last 6 months	Less than 1 h	1–3 h		3–5 h	5–10 h	10–20 h	20–40 h	More than 40 h
**How do you experience your POSITIVE EMOTIONS (for example: happiness, hope, joy, love)?**								
1: I experience them less vividly than before the pandemic	2	3: I experience them similarly as before the pandemic				4	5: I experience them more vividly than before the pandemic	
**How do you experience your NEGATIVE EMOTIONS (for example: sadness, anger, fear, disgust)?**								
1: I experience them less vividly than before the pandemic	2	3: I experience them similarly as before the pandemic				4	5: I experience them more vividly than before the pandemic	

### Procedure

The study was programmed in Javascript using PsychoJS and hosted on the Pavlovia hosting service. Participants always started with completing the CDS-29 questionnaire, which was then followed with the survey asking about lifestyle before and during the COVID pandemic. Questions were displayed individually and there was no possibility to return to previous questions. Participants were informed that they could withdraw from the study at any time by pressing ‘esc’. After completing the study, participants were debriefed via instructions displayed on the screen. They also received information about whether their score in CDS-29 was relatively high (if above 50) or low (below 20), and referred to a specific page for further information about depersonalization.

### Data processing and analysis

Data processing and analysis was conducted using custom scripts written in Python. Bayesian analyses were conducted using JASP 0.9.0.1.

For estimation of lifestyle habits before and during the lockdown we used (a) participants’ responses about the frequency of each activity during the last 6 months as indices of their lifestyle habits during the lockdown, and (b) how much the pandemic had impacted their lifestyle habits, as indexed by the difference between the frequency of each activity before and during the pandemic measured as the frequency of the activity during the last 6 months minus the frequency of the activity before the pandemic (positive values represent an increase in the activity during the pandemic).

Due to the fact that frequencies were measured on an ordinal scale, all analyses involving them were conducted using non-parametric statistical methods. Wilcoxon’s tests (W) were used in order to compare lifestyle behaviours before the pandemic with lifestyle behaviours during the pandemic. Kendall’s tau B (Tau B) was used in order to investigate the relationship between the participant’s estimations of the influence of lockdown on their life and the reported time they spent weekly on each life activity. We also used Kendall’s tau B to estimate the relationship between depersonalization (total CDS-29 score) and life activities (scores from the lifestyle survey). For these correlations we also reported the strength of the evidence in favour of the null hypothesis (BF01) and/or the strength of evidence in favour of the alternative hypothesis (BF10).

## Supplementary Information

Supplementary Information.
