# Supplementary material for: Zoomed out: digital media use and depersonalization experiences during the COVID-19 lockdown
Source: Sci Rep. 2022 Mar 10;12:3888. doi: 10.1038/s41598-022-07657-8 (PMC8913838; doi:10.1038/s41598-022-07657-8)
Supplement: Supplementary file 1 — Supplementary Information. [file 41598_2022_7657_MOESM1_ESM.docx]

**Supplementary Material 1: Impact of lockdown on the lives of individuals across different countries**

Although it is likely that at the time of administering our questionnaire (23 April and 8 May 2021) it is likely that all our participants had been significantly impacted by a lockdown in the last six months (we also excluded those who hadn’t experienced any lockdown in the last six months). Nonetheless, we carried out additional analyses to examine any between country differences with regards to the extent to which our participants felt impacted by the lockdown.

Firstly, we carried out a Kruskal-Wallis test using the respondents’ country of residence (we used the top five countries in terms of number of respondents who reside in that country: United Kingdom = 240; Poland = 94; Portugal = 68; Italy = 35; United States = 33) as an independent variable and reported impact of lockdown on life as the dependent variable. This analysis revealed a significant effect of country of residence, *H*(4) = 9.82, *p* = .044, d = .24

In order to verify that there were any differences between these countries with regards to reported impact of lockdown on life, we carried out Mann Whitney U (MWU) tests between each of these countries (Bonferroni corrected for 10 comparisons). We found no significant effects for any of these comparisons (see table below), thus no evidence that lockdowns impacted respondents residing in these countries differently. The lack of significant effects here could be due to lack of notable differences between the countries in terms of reported impact of lockdown on life, or due to the small sample sizes for some of the groups.

Overall, we take this as evidence that the effects we observe in our manuscript are unlikely to be explained by any between country differences in COVID-19 lockdown measures.

| **Comparison** | **MWU** | **p** | **d** |
| --- | --- | --- | --- |
| UK - Poland | 13001.5 | 0.23935147 | 0.24 |
| UK - Portugal | 7494.5 | 1 | 0.12 |
| UK - Italy | 4022.5 | 1 | 0.05 |
| UK - US | 4470.5 | 1 | 0.15 |
| Poland - Portugal | 2425 | 0.06197512 | 0.4 |
| Poland - Italy | 1326 | 0.78022063 | 0.3 |
| Poland - US | 1521 | 1 | 0.02 |
| Portugal - Italy | 1222 | 1 | 0.04 |
| Portugal - US | 1369 | 0.62109027 | 0.36 |
| Italy - US | 675.5 | 1 | 0.29 |

*Supplementary Table 1: Table displaying Mann Whitney U tests (Bonferroni corrected) between each of the top five countries in terms of number of respondents who reside in that country. This table displays the MWU statistic, p-value and effect size (Cohen’s d).*
